# Investigation of *OCH1* in the Virulence of *Candida parapsilosis* Using a New Neonatal Mouse Model

**DOI:** 10.3389/fmicb.2017.01197

**Published:** 2017-06-30

**Authors:** Katalin Csonka, Máté Vadovics, Annamária Marton, Csaba Vágvölgyi, Erik Zajta, Adél Tóth, Renáta Tóth, Csaba Vizler, László Tiszlavicz, Héctor M. Mora-Montes, Attila Gácser

**Affiliations:** ^1^Department of Microbiology, University of SzegedSzeged, Hungary; ^2^Biological Research CenterSzeged, Hungary; ^3^Faculty of General Medicine, Department of Pathology, University of SzegedSzeged, Hungary; ^4^Departamento de Biología, Universidad de GuanajuatoGuanajuato, Mexico

**Keywords:** *Candida parapsilosis*, neonatal, mouse model, intravenous infection, cytokines

## Abstract

*Candida parapsilosis* is an opportunistic human fungal pathogen that poses a serious threat to low birth weight neonates, particularly at intensive care units. In premature infants, the distinct immune responses to *Candida* infections are not well understood. Although several *in vivo* models exist to study systemic candidiasis, only a few are available to investigate dissemination in newborns. In addition, the majority of related studies apply intraperitoneal infection rather than intravenous inoculation of murine infants that may be less efficient when studying systemic invasion. In this study, we describe a novel and conveniently applicable intravenous neonatal mouse model to monitor systemic *C. parapsilosis* infection. Using the currently developed model, we aimed to analyze the pathogenic properties of different *C. parapsilosis* strains. We infected 2 days-old BALB/c mouse pups via the external facial vein with different doses of *C. parapsilosis* strains. Homogenous dissemination of yeast cells was found in the spleen, kidney, liver and brain of infected newborn mice. Colonization of harvested organs was also confirmed by histological examinations. Fungal burdens in newborn mice showed a difference for two isolates of *C. parapsilosis*. *C. parapsilosis* CLIB infection resulted in higher colonization of the spleen, kidney and liver of neonatal mice compared to the *C. parapsilosis* GA1 strain at day 2 after the infection. In a comprehensive study with the adult mice infection, we also presented the attenuated virulence of a *C. parapsilosis* cell wall mutant (*OCH1*) in this model. Significantly less *och1Δ*/*Δ* null mutant cells were recovered from the spleen, kidney and liver of newborn mice compared to the wild type strain. When investigating the cytokine response of neonatal mice to *C. parapsilosis* infection, we found elevated TNFα, KC, and IL-1β expression levels in all organs examined when compared to the uninfected control. Furthermore, all three measured cytokines showed a significantly elevated expression when newborn mice were infected with *och1Δ*/*Δ* cells compared to the wild type strain. This result further supported the inclusion of *OCH1* in *C. parapsilosis* pathogenicity. To our current knowledge, this is the first study that uses a mice neonatal intravenous infection model to investigate *C. parapsilosis* infection.

## Introduction

*Candida* species are common agents causing invasive diseases in neonates (Stoll et al., 2002; Benjamin et al., 2006; Chow et al., 2012). Although the incidence of invasive fungal infections among premature infants in neonatal intensive care units (NICUs) has decreased during the past decade (Aliaga et al., 2014; Kelly et al., 2015), candidiasis and infection-related death is still of major concern (Le et al., 2013).

Previously, epidemiological reports have demonstrated the difference in the range of infections caused by *Candida* species (Dotis et al., 2012; Juyal et al., 2013; Jordan et al., 2014). While *Candida albicans* is the leading cause of invasive candidiasis in general, children under the age of 2 are at greater risk of infections caused by *C. parapsilosis*, an emerging non-*albicans Candida* species (Dotis et al., 2012; Juyal et al., 2013; Jordan et al., 2014).

Neonatal animal injection methods have been in use for more than 30 years with the purpose of modeling newborn diseases (Billingham and Silvers, 1961; Pope et al., 1979; Domer, 1988; Chen et al., 1989; Rodewald et al., 1992; Tang et al., 1996; Genovese et al., 1999; Venkatesh et al., 2007; Trofa et al., 2011; Tsai et al., 2011). These models proved to be excellent for studying *Escherichia coli-* (Cox and Taylor, 1990), *Pseudomonas aeruginosa-* (Tang et al., 1996), *Listeria monocytogenes-* (Chen et al., 1989; Genovese et al., 1999), and *Streptococcus-* (Rodewald et al., 1992) caused sepsis in neonate mice and analyzing the virulence factors of these pathogens or the possible treatment of invasive diseases. Also, the neonate animal models have proven to be efficient to study newborn-related *Candida* infections (Pope et al., 1979; Domer, 1988; Venkatesh et al., 2007; Trofa et al., 2011; Tsai et al., 2011). Early studies laid foundations to establish systemic candidiasis in newborn mice by gastric inoculation (Pope et al., 1979; Domer, 1988). Using-2-day old mouse pups, the intraperitoneal route of injection was used to demonstrate the disseminated infection and assessing virulence properties of the different *C. albicans* strains, including comparisons of survival proportions or the extent of organ colonization (Tsai et al., 2011). Trofa et al. (2011) provided the first report of *C. albicans* and *C. parapsilosis* infection in a neonatal rat model. This research also demonstrated the crucial role of the secreted lipases in the virulence of these species. The study concluded that preterm rodents show higher susceptibility to *Candida* infections and confirmed the utility of neonatal animal models to characterize *C. parapsilosis* pathogenesis.

In newborn mice, gastric or intraperitoneal inoculation has previously been carried out as a route of infection to model systemic candidiasis (Pope et al., 1979; Domer, 1988; Venkatesh et al., 2007; Tsai et al., 2011). However, these models still have several limitations. The colony counts have shown considerable variation between the already mentioned inoculation methods. Gastric infection of infant mice led to rapid transmission of *C. albicans* cells from the gut to the liver, and less frequently to other organs such as the kidneys and spleen (Pope et al., 1979; Domer, 1988). Following peritoneal injection, strong spleen attachment occurred, either by direct contact with the organ or through lymphatic channels (Tsai et al., 2011).

Systemic infection models in neonatal mice are valuable tools for studying severe newborn diseases (Vizler et al., 1993; Kienstra et al., 2007; Glascock et al., 2011). In this study, we introduce a new intravenous neonatal mouse model of *C. parapsilosis* infection, which could help to understand the complications in the response of the premature immune system to the systemic fungal invasions.

## Materials and Methods

### Ethics Statement

All animal experiments were performed by national (1998. XXVIII; 40/2013) and European (2010/63/EU) animal ethics guidelines. The experimental protocols were approved by the Animal Experimentation and Ethics Committee of the Biological Research Centre of the Hungarian Academy of Sciences and the Hungarian National Animal Experimentation and Ethics Board (clearance number: XVI./03521/2011.) with the University of Szeged granted permission XII./00455/2011 and XVI./3652/2016 to work with mice.

### *Candida* Strains

*Candida parapsilosis* GA1 (SZMC 8110) (Gacser et al., 2005), CLIB 214 (SZMC 1560) (Laffey and Butler, 2005) wild-type strains and *C. parapsilosis och1*Δ/Δ (Perez-Garcia et al., 2016) and *C. parapsilosis och1*Δ/Δ+*OCH1* (Perez-Garcia et al., 2016) were used in this study. *Candida* cells were grown overnight at 30°C in liquid YPD medium (1% yeast extract, 2% peptone, and 2% glucose). Before experiments, cells were harvested by centrifugation, washed twice with PBS (phosphate-buffered saline; 137 mM NaCl, 2.7 mM KCl, 10 mM Na2HPO4, 2 mM KH2PO4; pH 7.4) and counted using a hemocytometer.

### *C. parapsilosis* Intravenous Infection

Timed pregnant BALB/c mice were obtained in the specific pathogen-free animal facility of the Biological Research Center (BRC, Szeged, Hungary, XVI./2015.). Mice were monitored to determine the date of the nativity. They received commercial mouse food pellets and water *ad libitum*. On a post-partum day 2, mouse pups were weighed (weight of pups was around 2,2-2,7 g depending on the number of pups in the same litter) and randomized within cages prior the injection with 1 × 10^7^ or 2x10^7^ yeast cells/20 μl of *C. parapsilosis* strains or with sterile PBS (control). Briefly, the injection of newborn mice was performed with a 1 ml insulin syringe with 30 G X 8 mm needle via the external facial vein of newborn mice. Bubbles in the suspension were eliminated to prevent potentially lethal air emboli. The vein was visualized by transilluminating the head with a light source placed on the opposite side. After injection, pups were examined daily. At the indicated time-points, pups were sacrificed via decapitation for the analysis of fungal burden, cytokine response and histology. Adult mice data were previously published (Perez-Garcia et al., 2016). 10–12 weeks-old male BALB/c mice were injected via lateral tail vein with 2 × 10^7^yeast cells/100 μl of *C. parapsilosis* strains or with sterile PBS (control). They were terminated at the same time-points as pups.

### Fungal Burden

For the colony counts, kidneys, spleens, livers, and brains were collected, weighed, and homogenized in sterile PBS (in the case of the kidney, in sterile PBS containing one Complete Protease Inhibitor Tablet (Santa Cruz Biotechnology Lot.: J1012) per 50 ml). Homogenates were used for determination of fungal burdens by colony counting after plating serial dilutions on YPD agar plates per tissue. The CFUs were counted after 48 h of incubation at 30°C and expressed as CFU/g tissue. (No colonies were recovered from samples from mice challenged with PBS alone.) The remaining homogenates were centrifuged at 3000 rpm, 4°C, for 15 min, and the supernatants were stored at –20°C until cytokine measurement.

### Cytokine Measurement

TNFα, IL-1β, IL-10, and KC levels were determined by commercial ELISA kits (R&D Systems) according to the manufacturer’s instructions. Cytokine levels were measured from kidney and liver of *C. parapsilosis*-infected (1 × 10^7^ yeast cells/20 μl) and PBS control mice after 2 and 7 days of infection. The concentration of each cytokine was determined in units of pg/ml, and then recalculated as pg/g tissue in the sample.

### Histology

Whole spleens, kidneys, livers and brains of PBS control and *C. parapsilosis*-infected (2 × 10^7^ yeast cells/20 μl) mice were fixed and kept in 4% buffered formalin until processed for histology. Fixed organs were sectioned and stained with periodic acid-Schiff (PAS) using conventional staining methods. Tissue sections were analyzed with a BX51 OLYMPUS or Zeiss Imager Z1 microscope.

### Statistical Analysis

Statistical analysis was performed using the GraphPad Prism 7 software. Differences in fungal burden were determined using the Mann–Whitney *U*-test. Unpaired *t*-test was used for analysis of cytokine measurements. Differences between groups were considered significant at *p*-values of <0.05. The majority of the experiments were performed at least twice and at least 4 mice/group/time point/experiment. Data are presented as means with standard errors of the means (SEM).

## Results

### Characterization of the Intravenous *C. parapsilosis* Infection of Newborn Mice

**FIGURE 1:**
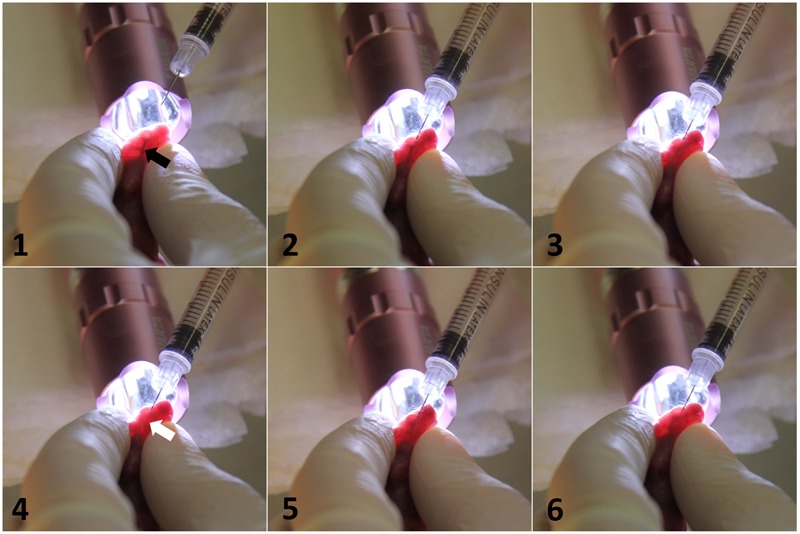
Intravenous infection of *C. parapsilosis* via injecting external facial vein of newborn mice. Transillumination of the head optimized the visualization of the blood vessel. Black arrow shows the target vein (1). After the insertion of the needle, the slow clearance of the vein from the blood is visible upon the expelling of the *Candida* suspension (2,3). White arrow shows the blanching of the vessel from blood (4). After the injection, the needle was withdrawn, and the blood returned to the vascular space (5,6).

In this research, we aimed to develop a new neonatal mouse model of invasive *C. parapsilosis* infection, using intravenous infection through the external facial vein. This method was originally developed for the adoptive transfer of hemopoietic cells (Billingham and Silvers, 1961). **Figure 1** shows the method of intravenous infection. Following transillumination of the pups’ head to aid the visualization of the vascular anatomy, the injection of newborn mice was performed via the external facial vein. BALB/c mouse pups were injected on post-partum day 2 with PBS (control) or wild-type *C. parapsilosis* (WT CLIB) (1 × 10^7^ yeast cells/20 μl).

**FIGURE 2:**
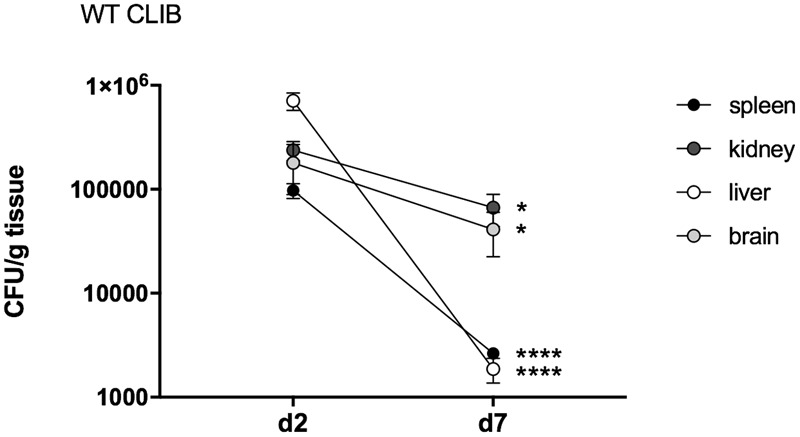
Fungal burden in neonatal mice after wild-type *C. parapsilosis* strain infection. CFU in spleen, kidney, liver and brain in newborn mice after 2 and 7 days of post-infection. At least *n* = 4 mice/group/experiment were infected with the 1 × 10^7^/20 μl of *C. parapsilosis* CLIB 214 (WT CLIB) wild-type strain. Results (mean ± SEM) are pooled data from two independent experiments. Not significant (ns), ^∗^*p* < 0.05 and ^∗∗∗∗^*p* < 0.0001 when the CFUs were compared between the two-time points of the analysis as determined by Mann–Whitney *U* test.

To monitor the course of the infection in this neonatal model, we included an early (day 2 post-infection) and a late (day 7 post-infection) time point examination. Fungal burdens were detectable in the examined organs (kidney, spleen, liver, brain) on day 2, indicating homogenous dissemination of *C. parapsilosis* cells. The highest number of CFUs was detected in the liver on day 2. Therefore, the injection via the external facial vein was not associated with higher fungal attachment to the brain compared to other organs. Following the course of the infection, decrease of the colony counts was detectable to day 7. On this day, the kidney showed the highest number of yeast cells, followed by the fungal burden in the brain, while the highest clearance was observable in the spleen and the liver (**Figure 2**).

**FIGURE 3:**
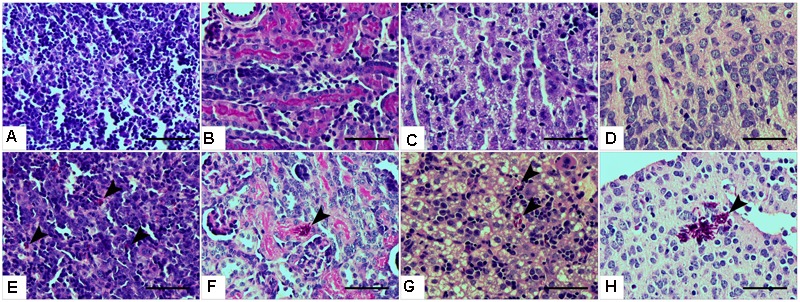
Histopathology during systemic *C. parapsilosis* infection in neonate mice. Sections were stained with periodic acid-Schiff (PAS) stain. **(A)** Spleen, **(B)** kidney, **(C)** liver, and **(D)** brain show the organs from PBS-injected mice. Fungal cells (indicated by arrowheads) are present in the organs of mice infected with the 2 × 10^7^/20 μl *C. parapsilosis* CLIB 214 (WT CLIB) (**E**, spleen, **F**, kidney, **G**, liver, **H**, brain) wild-type strain. All observations were performed at day 2 post-infection. Scale bar represents 100 μm.

Histopathological examinations also indicated the presence of *C. parapsilosis* yeast cells in all the examined tissues at day 2 post-infection, when the 2 × 10^7^ inoculation dose was used, showing the widespread colonization of the organs via the blood vessels (**Figures 3E–H**). However, no fungal cells were detected in the harvested organs during histology analysis at day 7 post-infection (2 × 10^7^), or at either time points in mice infected with 1 × 10^7^ *C. parapsilosis* cells (data not shown). Single cells of WT CLIB penetrated the different sites of the spleen (**Figure 3E**) and liver (**Figure 3G**). Colonization of the brain (**Figure 3H**) and the small blood vessels of the kidney was visible at day 2 post-infection (**Figure 3F**). The presence of fungal cells in the organs of the WT CLIB-infected mice was not associated with lesions or specific signs of immune cell infiltration. Of note, PBS-injected mice from the same litter showed no evidence of fungal cells or immune cell infiltrates in the examined organs (**Figures 3A–D**).

**FIGURE 4:**
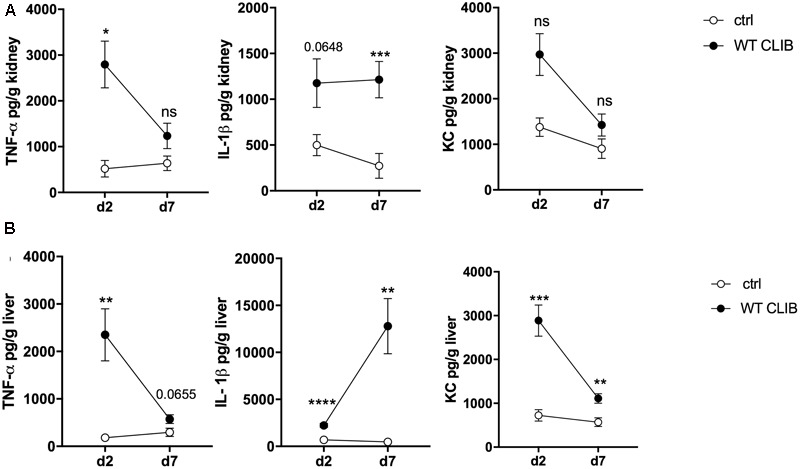
*In vivo* cytokine response in neonate mice after wild-type *C. parapsilosis* infection. TNFα, IL-1β, and KC level in the kidney **(A)** and liver **(B)** after 2 and 7 day of post-infection. Newborn mice were infected with the dose of 1 × 10^7^/20 μl *C. parapsilosis* CLIB 214 (WT CLIB) strain or with 20 μl of PBS (control group, ctrl). Results (mean ± SEM) are pooled data from at least two independent experiments (at least 4 mice/group/experiment). Not significant (ns), ^∗^*p* < 0.05, ^∗∗^*p* < 0.01, ^∗∗∗^*p* < 0.001, and ^∗∗∗∗^*p* < 0.0001 when it was compared to the control group and determined by unpaired *t*-test.

We also assessed the *in vivo* cytokine response in the colonized neonatal organs after 1 × 10^7^ dose of WT CLIB infection. TNFα, IL-1β, IL-10, and KC chemokine levels were measured from tissue homogenates of kidneys and liver, using enzyme-linked immunosorbent assay (ELISA). We could not detect these cytokines in spleen and brain homogenates. In the kidney, elevated TNFα level was found at day 2 post-infection stimulated by WT CLIB, and the quantity of this cytokine decreased at the later time point (**Figure 4A**). IL-1β was also induced at day 2 post-infection and its level remained elevated at day 7. Similar to TNFα, the secretion of KC was induced at day 2 post-infection, and a decrease in KC production was observed at day 7.

In the liver, high amount of TNFα was triggered by the WT CLIB at day 2 after infection. Similar to what was measured in the kidney, a decrease in the level of this cytokine was found at the later time point. At day 7 post-infection, an increase in the IL-β level was measured in this organ after WT CLIB injection. In comparison with the PBS control group, a rise in KC level was measured at day 2 after inoculation. Also, a reduction in the KC level was found at day 7 (**Figure 4B**). No significant IL-10 secretion was assessed from the kidney and liver samples after stimulation with WT CLIB (data not shown). The first set of analyses confirmed the applicability of this injection method in modeling disseminated candidiasis in neonatal mice.

### The Utility of the Newborn Mice Model to Investigate the Virulence Properties of *C. parapsilosis*

**FIGURE 5:**
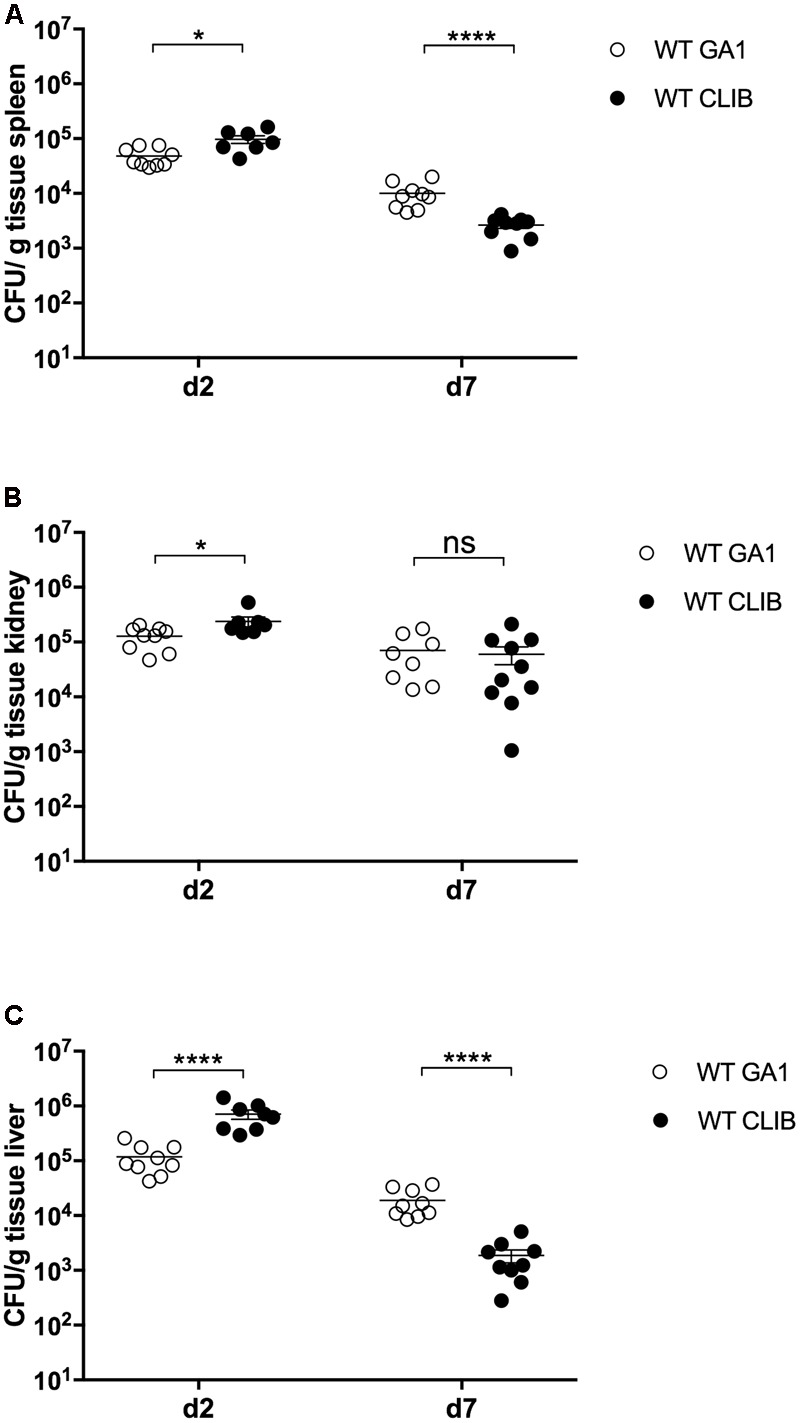
Fungal burden in neonatal mice after wild-type GA1 and CLIB *C. parapsilosis* strains infection. CFU in spleen **(A)**, kidney **(B)**, and liver **(C)** in newborn mice after 2 and 7 day post-infection. *n* = 4–5 mice/group/experiment were infected with 1 × 10^7^/20 μl of *C. parapsilosis* GA1 (WT GA1) and *C. parapsilosis* CLIB 214 (WT CLIB) wild-type strains. Results (mean ± SEM) are pooled data from two independent experiments. Not significant (ns), ^∗^*p* < 0.05 and ^∗∗∗∗^*p* < 0.0001 as determined by Mann–Whitney *U* test. Republished from Perez-Garcia et al. (2016).

We have previously reported that the GA1 and CLIB wild type *C. parapsilosis* strains elicit distinct host responses during *in vitro* experiments with murine and human macrophages (Toth et al., 2015). Following the inoculation of newborn mice with these strains (infection dose 1 × 10^7^), differences between the two strains were detectable in terms of fungal burden (**Figure 5**). At day 2 post-infection, the WT CLIB strain showed notably higher fungal loads in the spleen, kidney and especially in the liver, when compared to the WT GA1 strain. In contrast, 7 days after the infection, newborn mice showed higher ability to clear the WT CLIB strain from spleen and liver. However, no difference was found in the colony counts in the kidney.

**FIGURE 6:**
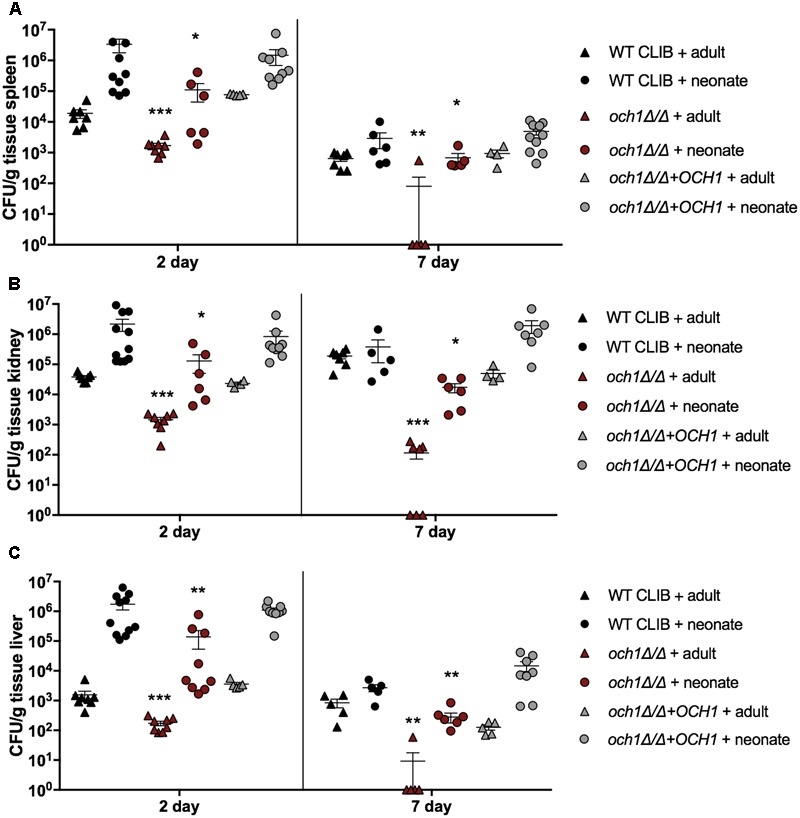
Comparison of fungal burden in the adult and neonate mice after the WT CLIB, *och1Δ*/*Δ*, and *och1Δ*/*Δ*+*OCH1* challenge. Newborn (× 10^7^/20 μl) and adult (2 × 10^7^/100 μl) mice were infected with the *C. parapsilosis* CLIB 214 (WT CLIB), the cell wall mutant (*och1Δ*/*Δ*) and the reintegrated (*och1Δ*/*Δ*+*OCH1*) strain. CFUs in spleen **(A)**, kidney **(B)**, and liver **(C)** at day 2 and 7 post-infection are shown. Results (mean ± SEM) are pooled data from two independent experiments (*n* = 5 adult mice/group/experiment, neonates at least 4 mice/group/experiment). Not significant (ns), ^∗^*p* < 0.05, ^∗∗^*p* < 0.01, ^∗∗∗^*p* < 0.001, and ^∗∗∗∗^*p* < 0.0001 when compared to the corresponding WT CLIB infected mice group as determined by Mann–Whitney *U*-test.

**FIGURE 7:**
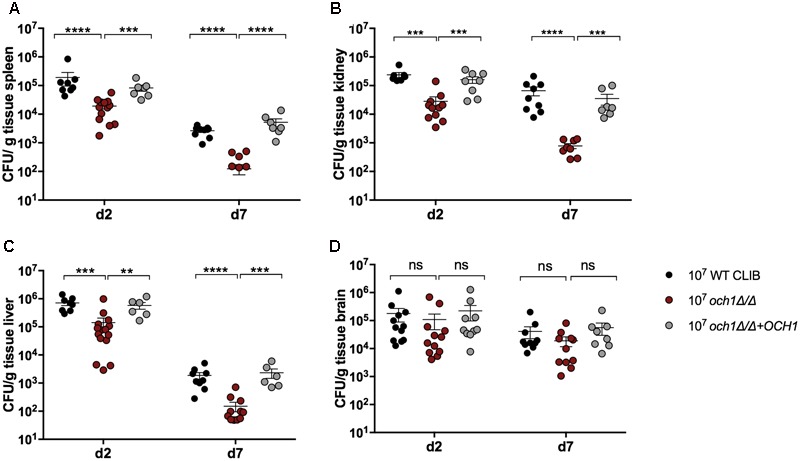
Comparison of fungal burden in neonate mice after the 1 × 10^7^ dose of WT CLIB, *och1Δ*/*Δ* and *och1Δ*/*Δ*+*OCH1* infection. Newborn (1 × 10^7^/20 μl) mice were infected with the *C. parapsilosis* CLIB 214 (WT CLIB), the cell wall mutant (*och1Δ*/*Δ*) and the reintegrated (*och1Δ*/*Δ*+*OCH1*) strain. CFUs in spleen **(A)**, kidney **(B)**, liver **(C)**, and brain **(D)** at day 2 and 7 post-infection are shown. Results (mean ± SEM) are pooled data from two independent experiments (*n* = 4 mice/group/experiment). Not significant (ns), ^∗^*p* < 0.05, ^∗∗^*p* < 0.01, ^∗∗∗^*p* < 0.001, and ^∗∗∗∗^*p* < 0.0001 when compared to the WT CLIB infected mice group as determined by Mann–Whitney *U*-test.

Next, we wanted to examine the neonatal immune response to a cell wall mutant *C. parapsilosis* strain (*och1Δ*/*Δ*) that was previously constructed in our laboratory (Perez-Garcia et al., 2016). This mutant lacks the *N*-linked mannan outer chain in the cell wall, and it has been shown to have a significantly attenuated virulence in a BALB/c mouse model of systemic infection (Perez-Garcia et al., 2016). We analyzed the fungal burden in newborn and adult mice after injection with the 2 × 10^7^ dose of *och1Δ*/*Δ* mutant or the wild-type WT CLIB or the reintegrated strain (*och1Δ*/*Δ*+*OCH1*). The virulence differences between the wild-type and the mutant strains were evident from the significantly decreased burdens in the spleen, kidney and liver in both adults and pups challenged with the *och1Δ*/*Δ* strain at 2 as well as at 7 day post-infection (**Figure 6**). Neonatal mice infected with the same dose (2 × 10^7^) of *C. parapsilosis* cells displayed similar trend in organ colonization in comparison with adult mice, as it was previously published (Perez-Garcia et al., 2016). However, while *och1Δ*/*Δ-*infected adult mice almost completely cleared the infection by day 7, fungal burdens in newborn mice were still high at this time point. Infection with the lower dose (1 × 10^7^) similarly showed the attenuated virulence of the cell wall mutant strain with the significantly decreased colony counts of the *och1Δ*/*Δ* in the spleen, kidney and liver compared to the WT CLIB. Interestingly, the fungal burdens in the brain of newborn mice were similar following WT CLIB, *och1Δ*/*Δ*, or *och1Δ*/*Δ*+*OCH1* challenge (**Figure 7**).

**FIGURE 8:**
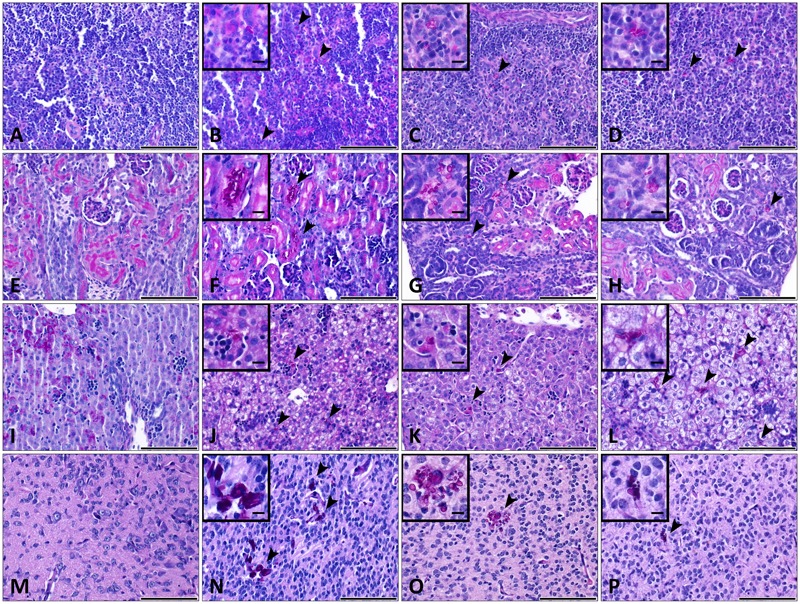
Histopathology during systemic WT CLIB, *och1Δ*/*Δ*, and *och1Δ*/*Δ*+*OCH1 C. parapsilosis* infection in neonate mice. Sections were stained with periodic acid-Schiff (PAS) stain. Fungal cells (indicated by arrowheads) are evident in the organs of mice infected with the *C. parapsilosis* CLIB 214 (WT CLIB) (**B** spleen, **F** kidney, **J** liver, **N** brain), the *och1Δ*/*Δ* (**C** spleen, **G** kidney, **K** liver, **O** brain) and the *och1Δ*/*Δ*+*OCH1* (**D** spleen, **H** kidney, **L** liver, **P** brain) strain. (**A** spleen, **E** kidney, **I** liver, **M** brain) Control organs from PBS-injected mice. All observations of the infected organs were performed at day 2 of 2 × 10^7^/20 μl dose of infection. Scale bar represents 100 μm.

Histopathological examinations indicated the presence of *och1Δ*/*Δ, och1Δ*/*Δ*+*OCH1* and wild-type *C. parapsilosis* yeast cells in the brain, kidney, liver and spleen of newborn mice at day 2 post-infection, when 2x10^7^ inoculum dose was used (**Figure 8**). The cell morphology of the strains were consistent with our previously published research (Perez-Garcia et al., 2016). The *och1Δ*/*Δ* cells were not able to develop pseudohyphae, and the rounded shape of the null mutant cells was distinguishable from the elongated cell form of WT CLIB or the *och1Δ*/*Δ*+*OCH1* strains during the histological analysis of the tissues. Single cells were spread in the spleen and liver of the three *C. parapsilosis* strains (**Figures 8B–D,J–L**). In the kidney, colonization by the fungal cells was observed around the small blood vessels (**Figures 8F–H**). Colonies were present in the small foci of the brain tissue (**Figures 8N–P**). However, the hematogenous spread of the mutant and the control strains was found in the tissues, specific signs of cellular response were generally not visible in the organs of the pups (**Figure 8**).

**FIGURE 9:**
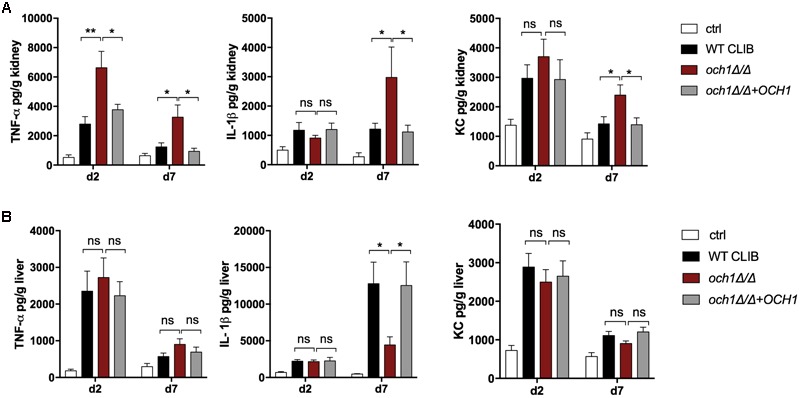
*In vivo* cytokine response in neonate mice after WT CLIB, *och1Δ*/*Δ*, and *och1Δ*/*Δ*+*OCH1 C. parapsilosis* infection. TNFα, IL-1β, and KC levels in the kidney **(A)** and the liver **(B)** after day 2 and 7 post-infection. Newborn mice were infected with the lower dose of 1 × 10^7^/20 μl with *C. parapsilosis* CLIB 214 (WT CLIB), the cell wall mutant (*och1Δ*/*Δ*), the reintegrated (*och1Δ*/*Δ*+*OCH1*) strains or with 20μl of PBS (control group, ctrl). Results (mean ± SEM) are pooled data from at least two independent experiments (at least 4 mice/group/experiment). Not significant (ns), ^∗^*p* < 0.05, ^∗∗^*p* < 0.01, when it was compared to the wild-type *C. parapsilosis* infected mice group determined by unpaired *t*-test.

According to the ELISA measurements, the lack of the *N*-linked mannan component in the *C. parapsilosis* cell wall resulted in altered cytokine response in newborn mice (infection dose 1 × 10^7^). In the kidney, *och1*Δ/Δ triggered significantly higher TNFα production compared to the wild-type and the reintegrated strain at the two time points of examination (**Figure 9A**). IL-1β and KC secretion was markedly increased in *och1*Δ/Δ-challenged newborn mice at day 7 post-infection compared to the WT CLIB, and the *och1Δ*/*Δ*+*OCH1*-challenged mouse groups. No significant differences were detected in the level of TNFα and KC in the liver of WT CLIB-, *och1*Δ/Δ-, and *och1Δ*/*Δ*+*OCH1*-challenged mice. However, the *och1Δ*/*Δ* strain induced significantly less IL-1β in the liver at day 7 post-infection compared to WT CLIB (**Figure 9B**). No difference was detectable in the levels of IL-10 from the kidney and liver of the different mouse groups (data not shown).

In conclusion, the neonatal mouse model proved to be suitable for the study of the virulence of different *C. parapsilosis* strains and compare the systemic *C. parapsilosis* infection in adult and newborn mice. In addition, our results further support the importance of the cell wall *N*-linked mannan in the pathogenesis of *C. parapsilosis*.

## Discussion

Mouse models have been widely used to study human diseases. The increased susceptibility of newborn mice and rat pups to systemic *C. albicans* infection has previously been reported (Pope et al., 1979; Domer, 1988; Trofa et al., 2011; Tsai et al., 2011). First studies demonstrated that gastrointestinal colonization and systemic disease could be achieved by gastric inoculation with *C. albicans* in a neonatal mouse model (Pope et al., 1979; Domer, 1988). In these studies, 5 to 6-day old mouse pups were infected and monitored for survival. Furthermore, tissue burden showed the colonization of spleen, kidney, liver and lung by *C. albicans* strains. Therefore, these early studies demonstrated that infant mice lend itself to study the host response against *Candida* infections. In 2011, new mouse model was developed by Tsai et al. (2011). In that model, mouse pups were infected with *C. albicans* by intraperitoneal route on post-partum day 2. Similarly, survival, fungal burden and histopathology were analyzed. They showed that intraperitoneal injection of *C. albicans* induced mortality in a dose-dependent fashion and led to disseminated disease as colonization of the spleen, kidney, liver, and lung were detected. Although this infection method resulted in the dissemination of the fungal elements to different organs, CFU counts were highly variable. Another neonatal animal model was described by Trofa et al. (2011), where 2–3 day old rat pups were infected with *C. albicans* and *C. parapsilosis* via intragastric, intraperitoneal and intravenous routes. In that study, colony counts demonstrated that lipase mutants *Candida* strains displayed lower virulence in the neonatal rats. In that experiment, also, *C. parapsilosis* infection resulted in overall lower fungal burden compared to *C. albicans*. Further investigation showed that the neonatal rat model proved to be suitable for the testing of antifungal prophylaxis with fluconazole, supported by significantly enhanced survival and weight gain after coinfection with *C. albicans* and *S. epidermidis* (Venkatesh et al., 2007). Taken together, these studies confirmed that neonatal rodent models are useful for studying *Candida* pathogenesis that may be specific to an immature host. Despite the importance of *C. parapsilosis* in neonatal infections, study of the *in vivo* immune response against *C. parapsilosis* has been hindered by the lack of appropriate animal models. The mouse intravenous challenge is well investigated and reproducible model to define *Candida* virulence (Arendrup et al., 2002). Therefore, here, we characterized a newborn mouse model of intravenous *C. parapsilosis* infection. The first part of our results revealed the utility of facial external vein injection as a novel method to induce systemic *Candida* infection in newborn mice. We observed widespread dissemination of the yeast cells in the spleen, kidney, liver and brain at day 2 and 7 post-infection during CFU determination and histopathological analysis. The benefit of the intravenous injection used in our study is the consistency in fungal burden measurements in the kidney, liver and spleen of newborn mice, indicating effective colonization of these organs. The fact that the route of the temporal vein injection was not associated with higher yeast attachment to the brain further supported the reliability of this model to characterize disseminated candidiasis in neonatal mice. Furthermore, ELISA measurements demonstrated that *C. parapsilosis* infection induced increased TNFα, IL-1β, and KC secretion in the kidney and liver in newborn mice. Therefore, the route of the injection and the features of this neonatal mouse model grant the opportunity to compare the susceptibility of neonate mice to the different *C. parapsilosis* strains and analyze the induced immune response in the adult and the neonatal settings during systemic infections.

In this research, we tested the utility of the neonatal candidiasis model to analyze the pathogenic properties of the different *C. parapsilosis* strains. Previously, it has been demonstrated that different isolates of this species trigger various host cell responses (Toth et al., 2015). It has been shown that macrophage migration is significantly enhanced toward WT CLIB than toward the WT GA1 strain. Furthermore, both murine macrophages and human PBMC-DMs require more time to engulf WT CLIB cells than WT GA1 cells. The altered cellular immune responses have been suggested to originate from potential differences in the cell wall structure or composition and fungal signaling molecules released by the two strains (Toth et al., 2015). In the present study, fungal burden results reflected the differences in the virulence properties of the two isolates. Compared to WT GA1, WT CLIB infection led to higher colonization of spleen, kidney, and liver at the early time point of infection. However, clearance by the neonatal host was more effective against the WT CLIB strain, as the WT GA1 showed higher tissue burden in the spleen and liver at 7 day after the infection. According to the study of Trofa et al. (2011), *C. parapsilosis* GA1 strain inoculation of rat pups did not induce mortality and the intragastric and intraperitoneal inoculation with 1 × 10^7^ cells of this pathogen led to complete clearance by the used host model organism on day 6. Similarly, during our experiments, infection of neonatal mice with the two *C. parapsilosis* isolates was not associated with mortality and the colonization showed decreased fungal burdens overtime. Therefore, these results contribute to a deeper insight into *C. parapsilosis* infection in the neonatal animal settings.

The *N*-linked mannosylation has recently been shown to play a role in the virulence of *C. parapsilosis* (Perez-Garcia et al., 2016). Disruption of *C. parapsilosis OCH1* results in morphological changes and decreased fungal load in the mouse model of systemic candidiasis. Our data support these results by the significantly reduced fungal burdens in the spleen, kidney and liver of newborn mice after even the lower dose (1 × 10^7^) or the higher dose (2 × 10^7^) of the *och1*Δ/Δ stimulus compared to the wild-type strain. No significant difference was detected in the colony counts in the brain between the wild-type and the cell wall mutant strain infected groups. *In vitro* studies with *C. albicans* demonstrated the invasion of brain endothelial cells by trans-cellular migration and the role of fungal invasins, Als3 and Ssa1 mediated trafficking to the brain (Jong et al., 2001; Liu et al., 2011). Therefore, our results indicate that the *N*-mannan component in the cell wall structure may not affect the mechanism of how *C. parapsilosis* enters and colonizes the brain of mice. Future research could target the investigations of how other *Candida spp*. invade brain tissue from the circulation.

During the histopathological analysis, yeast cells of the less virulent cell wall mutant, as well as the wild-type strain were present in the organs without specific damage of the tissues or visible cellular changes after the 2 × 10^7^ dose of the infection. This phenomenon likely relates to the route of infection and to the inoculation dose, and may also be influenced by the early developmental stage of the animal, as no evidence of yeast cells were detected at the lower dose of the infection.

Perez-Garcia et al. (2016) have shown that *C. parapsilosis N*- and *O*-linked mannans play different roles in host response than *C. albicans* mannans. *C. albicans* cells lacking *N*-linked mannosyl residues induced lower levels of inflammatory cytokines in human mononuclear cells and significantly decreased TNFα and IL-6 in kidneys in infected mice (Netea et al., 2006). In contrast, IL-6, IL-10, TNFα, and IL-1β production by human PBMCs was found to be higher following incubation with *och1*Δ/Δ compared to the wild-type *C. parapsilosis* strain (Perez-Garcia et al., 2016). Here, we found increased TNFα, IL-1β, and KC secretion in the kidney of *och1*Δ/Δ-injected neonate mice, which result correlates with the previous findings in human PBMCs (Perez-Garcia et al., 2016). The liver showed slightly elevated TNFα as well, but we found decreased IL-1β and KC level after the cell wall mutant infection. Preceding research with glycosylation mutant *C. albicans* strains reported the varying chemokine or cytokine responses between tissues (spleen and kidney) from infected mice (Castillo et al., 2011). Thus, despite the mentioned differences between the kidney and liver in our research, cytokine measurements stimulated by the *och1*Δ/Δ correlates with the significantly decreased fungal burden. As the neonatal host showed less susceptibility against the cell wall mutant strain, the higher KC chemokine and TNFα could explain the higher activation of the immune cells, which contributed to the more effective clearance of the *och1*Δ/Δ. Taken together, our model has proven useful for studying the role of the cell wall elements during the host-pathogen interactions and our findings further strengthened the importance of *N*-linked mannosylation in the virulence of *C. parapsilosis*.

## Conclusion

In our results confirm that injection via the external facial vein is a reliable and efficient method to induce disseminated *Candida* infection in newborn mouse. Therefore, for the future studies this novel model will be an adequate tool to compare the host responses of adult and newborn mice and will be useful for the analysis the pathogenic features of the different *Candida* strains in the neonatal host system. This model may also allow for testing of novel therapeutic strategies against systemic neonatal candidiasis.

## Author Contributions

AG, HM-M, and KC contributed to the conception of the study, HM-M, CVA, LT, CVI, and KC designed the study. KC, MV, RT, LT, and AM carried out experiments, AG, EZ, and KC analyzed the data. AG, AT, and KC wrote the main manuscript text, EZ, MV, and KC prepared manuscript figures. All authors reviewed the manuscript, contributed to the discussion and approved the final version.

## Conflict of Interest Statement

The authors declare that the research was conducted in the absence of any commercial or financial relationships that could be construed as a potential conflict of interest.
